# Sex on the Fly

**DOI:** 10.1371/journal.pbio.1000364

**Published:** 2010-05-04

**Authors:** Kira Heller

**Affiliations:** Freelance Science Writer, Oakland, California, United States of America

An American was sitting in a Paris cafe eating soup when a fly dropped into her bowl. “There's *un mouche* in my soup!” she exclaimed. A nearby waiter corrected her. “Non, Madame. *Une mouche*.” The American replied, “Wow, you French have good eyes!”

Although flies look pretty much alike to most people, to scientists working with *Drosophila melanogaster* fruit flies under a dissecting microscope, the differences between adult male flies and female flies are obvious. For starters, the abdomens of females are elongated and pointy, whereas those of males are more rounded and have a distinctive dark patch. In addition, males have sex combs—a fringe of blunt bristles on their first legs. However, in most tissues and organs, there are no discernable differences between the sexes. So does this sexually amorphic tissue actually “know” what sex it is? Carmen Robinett, Bruce Baker, and their colleagues address this question in their paper “Sex and the Single Cell. II. There Is a Time and Place for Sex” in this issue of *PLoS Biology*.

Like mammals, female *Drosophila* have two X chromosomes, and males are XY. In mammals, sexual differentiation depends on expression in gonadal tissue of the *SRY* gene, which resides on the male-specific Y chromosome. If *SRY* is expressed, gonadal tissue turns into testes. If it's not expressed, the tissue becomes ovaries. The newly formed gonads then secrete sex hormones, such as testosterone and estrogen, which have a major role in determining whether an organism develops male or female sexual characteristics.

Sexual differentiation is different in *Drosophila*, as Bruce Baker and Kimberly Ridge first described in a 1980 *Genetics* paper called “Sex and the Single Cell. I. On the Action of Major Loci Affecting Sex Determination in *Drosophila melanogaster*.” Instead of being told how to differentiate by hormones, the sexual identity of somatic cells in flies is specified cell autonomously (each cell specifies its own sex). The first step in the sex determination of cells is assessment of the ratio of X chromosomes to autosomal (non-sex) chromosomes. The ratio in XX cells activates a hierarchical cascade of regulatory genes, the first of which is *Sex lethal* (*Sxl*); in XY cells, the ratio keeps *Sxl* turned off in males. At the bottom of the hierarchy is the *doublesex* (*dsx*) gene; due to *Sxl*-regulated sex-specific splicing of *dsx* messenger RNA, different versions of DSX protein are expressed in male and female flies.

To find out if all of the somatic cells in *Drosophila* males and females are capable of sexual differentiation (i.e., express *dsx*), Robinett, Baker, and colleagues used a genetic technique that allowed them to visualize all of the cells expressing *dsx* in female and male flies. Surprisingly, they found that although *dsx* was expressed in nearly all cells in some tissues, in other tissues it was expressed in only a few cells, or in none at all. Thus, rather than being “all female” or “all male,” *Drosophila* are actually fine mosaics of sexually differentiated and undifferentiated cells.

The researchers' finding provides some insight into previous observations that in some cells and tissues, such as those of the genitalia, *dsx* regulated sex-specifically the expression of proteins that are used in non-sex-specific ways in other cells and tissues. Under the previously prevailing view that *dsx* was expressed in all cells, these findings had been puzzling. What these authors' current results suggest is that the patterns of regulation by *dsx* are simply reflecting the cellular pattern of *dsx* expression. They also found that instead of sexual differentiation being limited to adults, *dsx* was also expressed in certain larval tissues that might be used differently in males and females during pupation and young adulthood.

The fact that *dsx* isn't expressed in every cell means that the canonical pathway of *Drosophila* sex differentiation, in which the activity state of *Sxl* is the sole factor determining the sexual identity of somatic cells, may be due for some revision. In addition to the key regulatory role of *Sxl*, it seems that there's another layer of regulation in the sex differentiation hierarchy, in which temporal and spatial regulation of the transcription of sex determination genes, such as *dsx*, results in only some cells having the ability to respond to the activity state of *Sxl* by producing male- or female-specific DSX proteins.

This work has some far-reaching implications for the evolution of sex determination: different species of animals possess many of the same genes and share many similarities in how they regulate basic cellular and developmental process. Thus, it's not hard to imagine that similar mosaic patterns of sexual differentiation may occur in other animals with two sexes. Indeed, the authors of a recent *Nature* paper, titled “Somatic Sex Identity Is Cell Autonomous in the Chicken,” found that lateral gynandromorph chickens, in which one side of the bird has female characteristics and the other side has male characteristics, are a mosaic of ZZ (male) and ZW (female) cells, with ZZ cells predominating on the side with male characteristics and ZW cells on the female side. Unlike in mammals, where sex hormones influence the sexual identity of the entire organism, in chickens, as in *Drosophila*, sexual identity is almost entirely cell autonomous.

What evolutionary advantages might sexual identity mosaicism offer animals? Robinett, Baker, and colleagues suggest that particular cells and tissues may acquire the potential for sexual differentiation when it offers a selective advantage, but that universal sexual differentiation could negatively affect fitness; according to previously published research and the unpublished results of the authors, ubiquitous expression of male-specific DSX in all cells is detrimental to flies. Furthermore, the ability to deploy sexual identity regulatory genes in otherwise undifferentiated tissue may actually contribute to species diversification. These findings open up a new area in the field of sexual identity, and it will be interesting to see whether the mosaic nature of fly sex differentiation occurs in other animals, particularly those in which sexual identity is highly cell autonomous.

**Robinett CC, Vaughan AG, Knapp J-M, Baker BS (2010) Sex and the Single Cell. II. There Is a Time and Place for Sex. doi:10.1371/journal.pbio.1000365**

**Figure:**
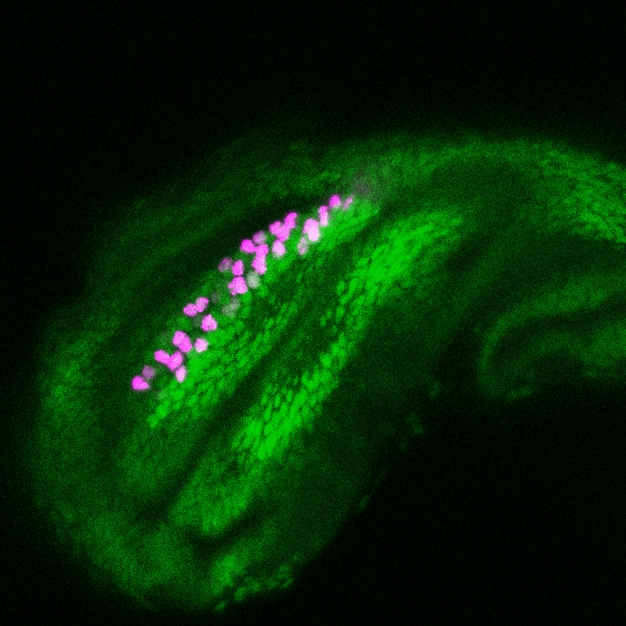
In the developing foreleg of *Drosophila*, *dsx* expression (magenta) is specifically activated in the region of the tibia, where it overlaps with a swath of *Sex combs reduced* protein (green).

